# Advancing insights into the neuroendocrine basis of socio-sexual interactions in mammals

**DOI:** 10.1210/endocr/bqag036

**Published:** 2026-03-30

**Authors:** Mona Masoumparast, Jean-Philippe Fiset, Nabil Nasri, Mauro S B Silva

**Affiliations:** Department of Obstetrics, Gynecology and Reproduction, Université Laval, Quebec City, QC GG1V 0A6, Canada; Reproduction, Maternal and Child Health (RSME) Axis, CHU de Québec-Université Laval Research Centre, Quebec City, QC G1V 4G2, Canada; Department of Obstetrics, Gynecology and Reproduction, Université Laval, Quebec City, QC GG1V 0A6, Canada; Reproduction, Maternal and Child Health (RSME) Axis, CHU de Québec-Université Laval Research Centre, Quebec City, QC G1V 4G2, Canada; INSERM U1215, Neurocentre Magendie, Université de Bordeaux, 33000 Bordeaux, France; Department of Obstetrics, Gynecology and Reproduction, Université Laval, Quebec City, QC GG1V 0A6, Canada; Reproduction, Maternal and Child Health (RSME) Axis, CHU de Québec-Université Laval Research Centre, Quebec City, QC G1V 4G2, Canada

**Keywords:** socio-sexual behavior, neuroendocrine circuits, sexual partner preference, gonadal steroid hormones, reproduction, developmental brain organization

## Abstract

Socio-sexual behaviors, a key aspect of mammalian biology, are governed by evolutionarily conserved neuronal circuits that control partner preference, sexual attraction, and attachment. This mini-review summarizes recent advances in understanding neuroendocrine pathways involved in various levels of socio-sexual interactions, from mating preferences to forming long-term sexual partnerships. We first briefly examine how prenatal hormone exposure shapes brain structures that later influence partner choices, with a particular focus on mechanisms driven by sex steroid hormones in rodent models. We also highlight some of the latest evidence showing how multimodal sensory cues activate neural circuits and neuroendocrine responses to initiate sexual behaviors. Finally, we examine how molecularly defined neuronal populations differently impact sexual performance and socio-sexual attachment in a sex-dependent manner. Some of the evidence presented here might have been overlooked and warrants greater attention to improve guidance and discuss future directions for our field.

Imagine this: you arrive at a crowded party, drawn to someone's laugh across the room with little enticing hints in the air. You look at them, and your brain subconsciously responds to the curve of their smile, the timbre of their voice, even the faintest trace of their perfume. Meanwhile, in a laboratory room, a mouse sniffs the air, detecting the pheromones of a nearby conspecific, and hypothalamic neurons are firing in patterns shaped by hormones, leading them toward their future sexual partner. Although worlds apart, both scenarios are governed by the same fundamental neurobiology: the social behavior network (SBN), an evolutionarily conserved web of brain regions that dictates socio-sexual preferences and behavior. First conceptualized by Sarah W. Newman and James L. Goodson (1, 2), the SBN integrates hormonal signals with sensory input to drive attraction, courtship, and mating. The current view of the SBN comprises several interconnected brain regions, including the preoptic area (POA), with particular emphasis on its medial part (MPOA), the ventromedial nucleus of the hypothalamus (VMH), the bed nucleus of the stria terminalis (BNST), the medial amygdala (MeA), the lateral septum (LS), the anterior hypothalamus (AH), the periaqueductal gray (PAG), and reward processing regions [eg, nucleus accumbens (NAc) and ventral tegmental area (VTA)]. This mini-review examines how these circuits are formed, functionally refined, and ultimately activated to guide partner choice and sexual behavior across mammals.

What neural mechanisms transform fleeting attraction into lasting preference? How do hormones sculpt the brain circuits that guide our most fundamental socio-sexual choices? This article addresses these questions through four key lenses: (1) the prenatal hormonal programming that lays the neural groundwork for future mate preferences, (2) the sensory pathways that converts pheromones, sounds, and sights into sexual attraction, (3) the neuroendocrine cells involved in sexual intercourse, and (4) the neurochemical bonds that elevate sexual encounters into lasting attachments. The neural mechanisms discussed here are probably conserved across mammalian species, emphasizing the importance of well-designed animal studies in advancing neuroendocrine research. It is important to clarify that this mini-review focuses solely on the neuroendocrine mechanisms behind sexual partner preferences, specifically the biological processes that drive attraction to male or female conspecifics. While human sexuality involves a complex interplay of biological, psychological, and social factors, our focus is limited to the hormonal and neural pathways that influence mate choice in mammalian models. We highlight recent progress in sensory integration, hypothalamic–pituitary–gonadal (HPG) axis regulation, and neuropeptidergic control of socio-sexual behavior, offering a clear yet comprehensive overview of this evolving field.

## Prenatal and prepubertal brain organization of future sexual partner preferences

Mate selection in mammals involves multiple levels of choice: conspecifics vs non-conspecifics, same-sex vs opposite-sex, physical traits, courtship presentation, age, and various other nuances. Extensive research has shown that mate preferences are primarily shaped by the organizational actions of gonadal hormones during early development and by the activational actions of gonadal hormones during pubertal and adult development in the socio-sexual brain pathways (3). Organizational actions of gonadal hormones are mostly referred to as prenatal programming of neural substrates and are considered the first stage in the development of future mate preferences, whether male or female biased. This is built upon the masculinization or feminization of neural patterns, which is driven by chromosomal sex and gonadal hormone exposure throughout prenatal and early postnatal development in mammals (3-7).

Masculinization refers to the organization of neural circuits that support male-typical behaviors, a process driven primarily by exposure to testosterone during critical prenatal and early postnatal periods (8). In parallel, masculinization occurs in parallel with defeminization, which denotes the suppression or elimination of neural pathways underlying female-typical behaviors because of the same androgenic (and estrogenic) hormonal exposure. Conversely, feminization refers to active gonadal steroid hormone–driven organization of the brain toward a female phenotype (9, 10), and the mere absence of a male phenotype does not constitute feminization. These processes are highly dependent on species and critically determined by the developmental window of gonadal steroid signaling (prenatal, early postnatal, or prepubertal). In-depth analyses of these concepts and their application in neuroendocrinology are provided in reviews elsewhere (8, 11-13). Below, we examine a well-established instance of anatomical dimorphism within the SBN and present new perspectives on the topic.

**Figure 1:**
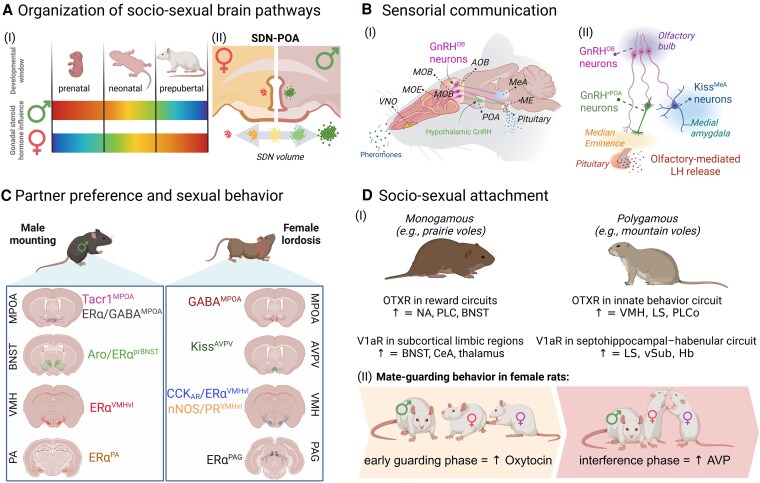
Proposed mechanisms modulating socio-sexual behaviors. (A) Developmental organization and sexual differentiation. (I) Hypothesized windows of sensitivity to gonadal steroid hormones in rodents, which are sex- and life-stage-dependent. In the spectrum bar, warmer colors indicate higher sensitivity, cooler colors lower sensitivity. (II) Prenatal androgen exposure in males and neonatal development in females shape the SDN-POA, producing male- or female-biased anatomical features that are associated with adult sexual preferences. (B) The case of GnRH^OB^ neurons and their control over socio-sexual behaviors. GnRH^OB^ neurons form reciprocal connections with the main and vomeronasal olfactory systems and with preoptic area GnRH neurons (GnRH^rPOA^). In males, GnRH^OB^ neurons influence opposite-sex odor preference, pheromone-induced hormone release, and sexual behavior via interactions with MeA kisspeptin neurons (Kiss^MeA^) and projections to the median eminence (ME). (C) Defining brain regions that modulate sexual behavior. Key regions involved in male and female sexual behavior in mice and rats, with coronal templates indicating the location of molecularly defined neuronal populations. Most of these regions positively modulate either sexual partner preference or sexual performance except GABA^MPOA^ neurons in females, which are known to attenuate sexual behavior. (D) Mechanisms supporting socio-sexual attachment. (I) Differential expression of oxytocin receptor (OTR) and vasopressin receptor (V1aR) in monogamous (prairie voles) vs polygamous (mountain voles), with arrows indicating higher receptor expression. (II) Mate-guarding in female rats: although polygamous, females display attachment behaviors to maintain partners, coordinated by oxytocin and vasopressin across two behavioral phases.

***The case of hormone-driven developmental masculinization:*** In altricial rodents, masculinization of behaviors is known to involve in situ testosterone conversion to estradiol by aromatase, and estrogen signaling directly affects the proliferation and communication of different neural cells within the male SBN during prenatal life (14-17). At the anatomical level, estrogen-mediated organizational effects are evident in male-biased regions such as the BNST, where prenatal expression of estrogen receptor α (ERα) is critical for establishing larger regional volume and male-typical behaviors, without altering HPG axis function in mice (17). A classic example of these organizational effects has been studied extensively in the central subdivision of the medial preoptic nucleus, also known as the sexually dimorphic nucleus of the POA (SDN-POA) (Fig. 1A). In rats, sheep, and primates, though this remains controversial in humans, males exhibit a larger SDN-POA volume than females (18-21). Discrete electrolytic destruction of the SDN-POA in the adult male rats does not affect copulatory behavior (22), but lesions of the regions encompassing the SDN-POA within the MPOA disrupt sexual partner preferences in male rats (23) and ferrets (24). The larger SDN-POA volume in males is largely determined by the number of Calbindin (Calb)-expressing GABA neurons under tight gonadal hormone action, primarily estrogens and testosterone, during early postnatal life (25). In mice, Calb-expressing GABAergic neurons in the POA are masculinized by estradiol during the early postnatal period (PND 1-5) (26). While estradiol may contribute to the neuronal population aggregates, prenatal androgens may dictate their projection pattern. Novel evidence shows that Calb SDN neurons are constituted mostly by two subpopulations: a VTA-projecting group (Calb^SDN-VTA^) and a medial POA efferent (Calb^SDN-MPOA^) (27). Calb^SDN-VTA^ neurons are exclusively found in males, whereas Calb^SDN-MPOA^ neurons are found in both sexes, a feature determined by the exposure to testicular androgens during both perinatal and peripubertal periods (27). The VTA is home to dopaminergic (DA^VTA^) neurons of the mesocorticolimbic dopamine system and a critical region governing motivation and reward-driven goals, such as social behaviors (28, 29). Barradas-Moctezuma and colleagues have recently demonstrated that neonatal gonadectomy disrupts female-directed partner preference and reduces the size of the SDN-POA in adult male rats, as expected. However, Pavlovian conditioning involving cohabitation with receptive females, under the influence of a dopamine D2 receptor agonist (quinpirole), restores social preference in these males for females, and normalizes SDN-POA size, even without testosterone supplementation (13). Dopaminergic mechanisms and learned social experiences may offset early androgen deprivation in adult partner preference and hypothalamic sexual dimorphism, but it is unclear what ensures plasticity in prenatally organized SDN-POA neurons.

***The case for whether developmental feminization truly occurs:*** In developmental neuroendocrinology, a prevailing view holds that the female hypothalamus undergoes a default developmental trajectory in the absence of androgen signaling or estrogenic actions derived from local aromatization during prenatal and neonatal life, a concept largely shaped by classic studies in rats and mice. Although adjudicating this debate is beyond the scope of the present review, we highlight a few examples suggesting that hypothalamic female development may not be solely a passive process. A recent study suggests that microglia-mediated phagocytosis plays a key role in determining the SDN-POA volume in females. Researchers found that between postnatal day (PND) 8 and PND 12 in rats, there is a consistent increase in the overall microglia population within the MPOA in both sexes (30). However, females exhibit a higher number of phagocytic-type microglia during this developmental window. Pharmacological or immunogenic inhibition of microglial activity during this critical neonatal period is sufficient to increase SDN volume in females, even in the absence of androgen treatment (30). Intriguingly, neonatal blockade of phagocytic microglia does not prevent adult female-like SDN-POA size but reduces adult olfactory preference for the opposite sex in females, a behavioral change linked to reduced neuronal activation within this nucleus (30). Do estrogens have an active role in shaping female hypothalamic development? Aromatase knockout (ArKO) female mice, therefore unable to convert testosterone to estradiol during development, subjected to ovariectomy and estradiol supplementation in adulthood, still display severe sexual receptivity and impaired sex-directed olfactory investigation (31). Markedly, administering estradiol between PND15 and PND25, before puberty can restore adult sexual preferences and lordosis behavior. In contrast, administering estradiol during the early postnatal period (PND5 to PND15) does not have this effect (32). It is also important to note that previous epigenetic studies in female rats and mice have shown that during neonatal life, estradiol actively prevents the development of a male-like DNA methylation pattern and the expression of male-like sexual behaviors (9). These postnatal and prepubertal molecular changes challenge the traditional view of organizational effects underlying female socio-sexual behaviors. For example, in males, a neonatal testosterone surge masculinizes neuronal populations in the anteroventral periventricular (AVPV) nucleus of the hypothalamus, including kisspeptin neurons (33, 34). In contrast, in females, this same kisspeptin population develops and undergoes organizational maturation primarily during the prepubertal life around PND25 in mice (10). Therefore, future research should clarify whether the differences in when key neuroendocrine cell groups are organized occur only in rodents or also appear in other mammals and how estradiol may play an active role in feminizing various behaviorally relevant neural pathways.

## How does the brain sense the partner's presence?

Mammals rely on multimodal sensory cues to identify mating partners, conveying bits of social information to potential sexual partners. Among these sensory systems, olfaction is primarily used in socio-sexual contexts to detect pheromones and sex-related volatile molecules that attract mating partners. The rodent olfactory system comprises two parallel pathways: the main olfactory system and the accessory olfactory system. The main olfactory bulb (MOB) receives input from the main olfactory epithelium (MOE) and projects to regions such as the piriform cortex and cortical amygdala, supporting odor perception. The accessory olfactory bulb (AOB), which processes pheromonal signals from the vomeronasal organ (VNO), first projects to the MeA, and information flows to BNST, mPOA, VMH, VTA, and NAc (35-39). Early studies demonstrated that the MOE plays a central role in the cognitive processing of mate recognition, while the VNO primarily drives neuroendocrine responses following exposure to sex-related olfactory cues [see review by Kelliher (40)]. By comparison, the human VNO is vestigial and lacks the genetic components necessary for pheromone signal transduction, such as the transient receptor potential cation channel subfamily C member 2 (TRPC2), suggesting that any pheromone-related responses are likely rerouted through the MOE pathway (41).

Accumulating evidence indicates that sex-biased sensory processing can be modulated by adult social environment. In mice, prolonged separation between sexes induces notable changes in olfactory receptor repertoires in both males and females (42). This may be due to differences in exposure to opposite-sex odors, since animals separated by sex mainly encounter odors from the same sex and themselves, while mice in mixed-sex groups experience a broader range of olfactory cues. This sensory difference seems to follow a “use-it-or-lose-it” mechanism: olfactory sensory neurons with specific receptors decrease in response to constant stimulation and increase when deprived of their ligands (42). Olfactory perceptual learning has been linked to adult neurogenesis in the rodent olfactory bulb, which can be stimulated by opposite-sex pheromones, thereby enhancing socio-sexual behaviors (43, 44). Blocking mouse neurogenesis with cytosine arabinoside (AraC) greatly reduces odor discrimination by enrichment, indicating adult neurogenesis is required for olfactory perceptual learning (45). Estradiol-deficient ArKO female mice show normal newborn cell growth in the olfactory bulb and odor discrimination, but have difficulty learning familiar male odors (46), which may contribute to their socio-sexual impairments (31). Conversely, studies in male mice demonstrate that testosterone modulates adult neurogenesis, activity within the AOB pathway, and olfactory preference, as evidenced by observations in testosterone-deficient Sema7a-KO mice. In both instances, it is not yet established whether these deficits result from a lifelong deficiency of gonadal steroid hormones. Furthermore, the mechanisms by which adult hormone levels might facilitate the recovery of olfactory learning through neurogenesis remain to be fully understood.

Reproductive behavior relies on the seamless coordination of sensory input and hormonal output, and recent findings illuminate how olfactory cues may directly affect the HPG axis. Gonadotropin-releasing hormone (GnRH) neurons are traditionally recognized for orchestrating reproductive function from the hypothalamus in the HPG axis, and their developmental journey begins in the embryonic nose (47-49). Although most of these neurons migrate to the septal and hypothalamic regions, we recently identified a significant subset of approximately 20% of these cells that remain in the olfactory bulb (OB GnRH) in mice (50). We found that murine OB GnRH neurons establish bidirectional connections with both MOE and VNO and the brain's neuroendocrine centers, such as the POA and median eminence (ME). In males, they are crucial for appropriate opposite-sex odor preferences, pheromone-driven hormone release, and optimal sexual performance (50). These behavioral responses are partly mediated through communication of OB GnRH neurons within the AOB with Kisspeptin neurons in the MeA, while the control over gonadotropin secretion occurs via direct OB GnRH neuronal projections to the ME and interaction between OB GnRH and the classical hypothalamic GnRH neuronal populations (50). We could also identify OB GnRH neurons in the neonatal and adult human brain (50), and we speculate whether they could be the long-awaited link that provides an MOE-VNO neural mechanism for aligning sexual behavior with reproductive endocrine readiness (Fig. 1B). Future studies may clarify their possible role in influencing olfactory and socio-sexual behaviors in females, which remains unknown.

Sex-related olfactory cues intertwine multimodal signaling to improve socio-sexual communication. Male mice dynamically modify their courting behavior based on combinations of female olfactory and vocal signals. Specifically, when both female urine and ultrasonic vocalizations (USVs) are presented together, males display a more elaborate courtship response, spending more time investigating and producing more USVs, compared to any other signal combination (51). Female urine alone increases male investigation, while female vocalizations without accompanying scent seem to have little effect (51). Thus, female olfactory cues provide context that helps males interpret potentially ambiguous female vocalizations, leading to enhanced courtship effort when both modalities are present. Male rodent USVs are under the modulation of testicular androgens, as castration decreases socio-sexual vocalizations, which are restored upon testosterone supplementation (52, 53). The neural substrates where testosterone acts to control these female scent-induced vocalizations are thought to involve olfactory pathways to the MeA and subsequent information toward the mPOA and BNST. However, among these regions, the mPOA is the site of final expression of sex-directed USVs, which is modulated by androgenic actions (54). Emerging data show that male USV production is driven by vesicular GABA transporter neurons in the MPOA (GABA^MPOA^) even when a female stimulus is absent (55). The male vocalization circuitry also appears to integrate modulatory inputs from other neuropeptidergic systems. For instance, the fine-tune control of male USVs may receive inputs from arginine vasopressin (AVP) neurons from the BNST (AVP^BNST^). Researchers used a viral-mediated shRNA approach to reduce AVP synthesis in AVP^BNST^ neurons. Following this intervention, male mice exhibited decreased USVs and copulatory attempts, whereas female mice did not show changes in social communication or behavior when AVP expression was absent (56). Female mice typically display less USV during socio-sexual contact, and one way to enhance USV emission and enhance same-sex social investigation is to subject them to a brief social isolation period. Recent findings show that POA neurons are required for these same-sex social interactions and USVs in female mice, whereas a corresponding population of male POA neurons is required for the display of opposite-sex social interactions and USVs (57). In female rats, multisensory stimuli of multiple modalities are also key for choosing an opposite-sex sexual partner and enhancing receptivity (58); yet, it remains under investigation the contribution of various neuroendocrine systems to coupling different sensorial modalities.

## When neuroendocrine circuits are ready for sex

While an in-depth review of mammalian neuroendocrine circuits is beyond this article's scope and recently reviewed by Mei et al (59), we will outline recent developments that have expanded our understanding of these complex neural pathways. In male mice, the activation of GABAergic neurons expressing estrogen receptor alpha (ERα) in the MPOA promotes robust sexual behavior in the presence of auditory cues (60), while tachykinin receptor 1 (Tacr1)-expressing neurons in the same area drive sexual mounting toward females and even inanimate objects (61). This generalized arousal, driven by Tacr1 neurons in the MPOA (Tacr1^MPOA^), modulates partner-specific responses through inputs of substance P (encoded by the *Tac1* gene), the strongest Tacr1 agonist, from BSNT neurons that recognize a conspecific's sex through differential activation patterns (61) and express ERα (62). Optogenetic stimulation of Tacr1^MPOA^ neurons also reduces the post-ejaculatory refractory period (inhibitory phase), enabling males to ejaculate again during mating (61). These findings align well with seminal work demonstrating that lesions of the BNST impair both anogenital investigation (appetitive phase) and copulation (consummatory phase); conversely, lesions of the MPOA affect only copulation, without changes in anogenital investigation in rodents (63, 64). We can now place that the BNST acts as the socio-sexual gate for sensorial pathways to sexual performance-promoting hypothalamic neurons. In the principal component of the BNST of naive male mice, neurons expressing aromatase (Aro^prBNST^) can respond to the presence of conspecifics through vomeronasal pheromone detection and, through different neuronal activation patterns, can distinguish whether they will engage in sexual mounting toward females or fighting with males (65). While aromatase expression suggests there is a local source of estradiol, it is still unclear how aromatase activity, estradiol production, and ER signaling contribute to the regulation of male sexual behavior by Aro^prBNST^ neurons. Another important neuroendocrine hub driving male sexual behavior was formerly identified in the VMH, where ERα+ and progesterone receptor-positive (PR+) neurons may trigger copulatory initiation (66, 67), which contributes to this behavior through projections from ERα neurons in the posterior amygdala (PA) to the MPOA (68) (Fig. 1C).

In females, four regions roadmap the flow of information leading to sexual behavior: AVPV, VMH, PAG, and MPOA. All these distinct regions receive the fluctuating actions of estrogens and progesterone throughout reproductive age to coordinate their role during appetitive, consummatory, and inhibitory phases of female sexual behavior. In the AVPV, kisspeptin neurons (Kiss^AVPV^) are activated by detecting male odors through the VNO pathway, direct sexual partner preferences, and are essential for the proper display of sexual lordosis in female mice (69). Kiss^AVPV^ neurons are central triggers of ovulation and might be the point of convergence between olfactory, neuroendocrine, and sex brain pathways to coordinate female reproduction (70, 71). AVPV neurons receive strong input from progesterone receptor (PR)-expressing neurons in the VMH, which exhibit increased cellular and functional connectivity during estrus (72). However, it remains unknown whether the postsynaptic cells are Kiss^AVPV^ neurons. Sex steroid-expressing neurons (ERα and PR) of the VMH have long been known for their key role in female sexual behavior. The ventrolateral part (VMHvl) is densely populated by neurons co-expressing ERα and PR, which are required for female sexual receptivity (67) and are active during mating interactions (72). Molecular and functional intersectional manipulations have identified a singular cluster within the ERα^VMHvl^ neurons expressing the cholecystokinin-a receptor (CCK_AR_^VMHvl^) that are required for female sexual behavior in mice and project to the AVPV (73). Remarkably, chemogenetic or optogenetic activation of CCK_AR_^VMHvl^ neurons in non-receptive female mice promotes sexual partner preference and receptivity toward males, while reducing sexual rejection and aggression (73). We further demonstrated that the chemogenetic inhibition of nNOS^VMHvl^ neurons robustly impairs female mouse sexual behavior, recapitulating a PCOS-like condition, and supplementation with nitric oxide (NO) is sufficient to rescue normal sexual behavior (74). A dense number of VMH^ERα^ neurons project to the PAG (75, 76), which seems to be the excitatory site integrating hypothalamic inputs to downstream pathways, such as the medullary reticular formation, to drive lordosis in female rats (75). The molecular identity of these downstream PAG neurons remains unclear, although some reports indicate that ERα^PAG^ neurons might play a role in female sexual behavior (76). Following lordosis and male ejaculation, female rodents typically decrease sexual motivation and receptivity. Lesions of the MPOA facilitate the display of consecutive lordosis (77), suggesting an inhibitory role of this region for female sexual behavior. Using the targeted recombination in active populations technique, brain-wide mapping of neural responses and calcium imaging, researchers have recently identified that GABA^MPOA^ neurons represent the decrease of female receptivity after male ejaculation (78). They also demonstrated that activation of GABA^MPOA^ neurons displays persistent activity after the onset of male ejaculation and promotes the suppression of female sexual motivation (78) (Fig. 1C).

## Building the socio-sexual attachment

Socio-sexual attachment might be the final stage in sustaining sexual partner preferences. In the context of this article, we define socio-sexual attachment as the enduring affective bond that arises from sexual activity extending into the post-copulatory period, without necessarily necessitating a strong emotional or commitment-based relationship. Among the widely researched neuroendocrine mediators of this attachment, oxytocin and AVP orchestrate affiliative behaviors and pair bonding through their action in hypothalamic regions, including the NAc, amygdala, and POA. In monogamous prairie voles (*Microtus ochrogaster*), oxytocin receptor (OTXR) density is higher in reward-related circuits (like the NAc, prelimbic cortex, and BNST) than in polygamous montane voles (*Microtus montanus*) (79). In the latter, OTXR is more concentrated in key components of the innate control of sexual behavior (VMH, LS, and posterior cortical amygdaloid nucleus [PLCo]) (79). This initial neuroanatomical characterization laid an important foundation for subsequent studies showing that central OTXR signaling mediates mating-induced partner preference formation by activating sensory and reward systems (80, 81). Neuroendocrine research using prairie voles has shown that forming monogamous bonds through partner preference relies on oxytocin signaling, and genetic variations of the OXTR in different brain areas influence social attachment and its associated rewards (82-84). These former studies were recently challenged by the demonstration that CRISPR-induced complete knockout of OTXR does not affect pair-bonding, fertility, and parental care in both sexes (85). The differences observed might result from pharmacological and adult viral interventions used in previous studies, as opposed to the lifelong absence of oxytocin signaling in CRISPR-induced OTXR-KO animals, which could trigger compensatory changes in other neuroendocrine systems. Alternatively, it may be necessary to refine OTXR ligands in pharmacological research to minimize crosstalk between pathways involved in pair-bonding.

The distribution pattern of the AVP receptor V1a is also different between these two species. Monogamous prairie voles have high V1aR density in the BNST, thalamic regions, and central nucleus of the amygdala, while polygamous montane voles present high V1aR density in the LS, ventral subiculum, and habenula (86). Of note, most differences between the species were not influenced by sex for both neuropeptides. A significant finding demonstrated that enhancing the expression of V1aR in the ventral forebrain of promiscuous male vole species via viral vectors promotes the development of sexual partner preference (87) (Fig. 1D).

Rodent species used for neuroendocrine research are mostly non-monogamous, which is partially due to the absence of a robust social memory to establish a single affiliative preference. Laboratory mice (*Mus musculus*) and rats (*Rattus norvegicus*) tend to prefer novel conspecifics, contrary to prairie voles and California mice (*Peromyscus californicus*), which can form selective partner preferences and social bonds (88). To artificially establish socio-sexual attachment in C57Bl/6J male mice, researchers observed that chemogenetic activation of a brain pathway from the paraventricular nucleus (PVN) of the hypothalamus to the CA2 in the hippocampus promotes partner preference toward a familiar female rather than a novel female (89). This might be linked to a known neuroendocrine pathway of PVN Arginine-vasopressin (AVP^PVN^) neurons acting on AVP 1b receptor (V1bR)-expressing pyramidal neurons in the CA2 (AVPr^CA2^), which modulate conspecific-dependent social discrimination and memory (90, 91). Thus, species-specific differences in AVP actions within brain regions involved in social memory, such as the hippocampus, may define how sexual partner preferences are translated into enduring social bonds.

Although female rats are naturally polygamous, they can display mate-guarding behavior, where a paced mating paradigm induces a preference for and protection of their paired male against mating with other females (92). These females also present more neuronal activation in oxytocin neurons, within the PVN and SON, and AVP neurons within the SON in response to the presentation of their chosen male rat (93). In addition, peripheral administration of both oxytocin and AVP to female rats enhances the acquisition of this socio-sexual attachment, in which each neuropeptide modulates different modules of the guarding behavior: Oxytocin contributes to the early phases of the mate-guarding display (eg, female remains around the chosen male so she can receive more mating attempts when a competitor female is around), whereas AVP acts during the interference phase (eg, female rat imposes its body between the chosen male and the competitor female, blocking their prospective sexual encounter) (93) (Fig. 1D). The development of mate-guarding behavior in female rats appears to be closely controlled by epigenetic mechanisms, as blocking histone demethylation prevents the formation of this attachment strategy (94).

Researchers have recently observed that socio-sexual interactions, regardless of whether mating occurs, induce proliferation and neuronal differentiation in an in vitro neurosphere system derived from the subventricular zone of prairie voles (SVZ) in both sexes (95). The SVZ is one of the primary neurogenic niches in the adult mammalian brain, located along the lateral walls of the lateral ventricles. It harbors neural stem and progenitor cells capable of generating new neurons and glial cells throughout life. These newly formed cells can migrate via the rostral migratory streams and contribute to brain plasticity and repair upon stimulation or injury under the influence of gonadal steroid hormones in both sexes (96). Social bond was associated with an increase in the number of mature neurons and a decrease in glial niches in neurospheres, which appears to be dependent on growth factors, sex steroid hormones, and oxytocin signaling (95). This evidence suggests that social-sexual interactions promote long-lasting molecular and cellular changes in neurogenic niches in the brain, and it remains to be investigated how newly generated cells can integrate into existing circuits and contribute to the neuroendocrine control of sexual partner preferences.

Social deficits, such as those caused by isolation or neurodevelopmental disorders, can significantly disrupt sexual bonding and performance. In this context, a recent study importantly addressed the link between male rats subjected to social isolation and the emergence of sexual aggression toward female conspecifics (97). This study used a modified version of the Sexual Aggression Test (SxAT), in which male Wistar rats are exposed to an intruder before the exposure to a non-receptive female (98). Researchers observed that by disrupting the hypothalamic–pituitary–adrenal (HPA) axis through prolonged isolation, male rats displayed substantial increases in offensive behaviors, such as forced sexual mounting, threats, and attack durations toward females (97). These behavioral changes were accompanied by neuroendocrine shifts, with serum AVP and corticosterone levels positively linked to aggression severity, while serotonin and oxytocin levels showed consistent negative correlations across both paradigms (97). Neurochemical receptor analyses demonstrated that social isolation mostly influenced the expression of AVPR1A and OXTR in both the prefrontal cortex and hippocampus (97). Briefly, isolation and aggression led to higher AVPR1A expression, while group-housed rats facing aggression showed lower OXTR expression, implying oxytocin may help regulate social stress (97). Overall, the findings reveal a complex and dynamic interaction among environmental factors (eg, housing conditions), neuroendocrine signaling, and sexually aggressive behavior.

## Conclusions and the road ahead

The neuroendocrine mechanisms that regulate socio-sexual attraction, partner selection, and attachment in mammals are intricate, and we are taking initial steps to understand this complexity. This review has highlighted only a few new findings showing how early-life hormone exposure shapes neural substrates for future mate preferences, how sensory cues are integrated into neuroendocrine pathways to direct sexual behavior, and how neuropeptides strengthen socio-sexual bonds in classical rodent models. We recognize that a more comprehensive review of the present research topic is needed; thus, we direct readers to specialized reviews on related topics elsewhere (8, 59, 99-103). Despite notable progress, several important questions remain unanswered, opening exciting opportunities for future research.

The precise gonadal hormone-mediated epigenetic processes, such as DNA methylation and histone modification, which are established during early development and around the onset of puberty to influence or maintain socio-sexual preferences later in life, remain insufficiently characterized. Future studies may examine the transmission of these dynamic epigenetic changes into adulthood, and how factors such as social experiences or hormonal shifts influence sexual attraction and socio-sexual attachment. Within the sensory integration helm, we still need further investigations into how different sensory modalities interact hierarchically to drive attraction. Most research has focused on heterosexual behaviors, leaving gaps in understanding the mechanisms of same-sex preferences. Human neuroimaging studies integrating hormonal and genetic data could bridge translational gaps in sexual orientation research. Comparative studies across species with diverse mating systems of monogamy, polygamy (polygyny, polyandry, and polygynandry), and promiscuity could help identify both conserved and divergent neural mechanisms underlying these behaviors. Such investigations may offer valuable insights not only into the biological roots of human socio-sexual behaviors but also into the development of more effective conservation strategies for endangered species. Disorders like hypoactive sexual desire disorder and PCOS-related sexual dysfunction accentuate the need for further investments in the field toward novel neuroendocrine therapies. From different angles and at the intersection of hormones, neural circuits, and lived experience lies a revolutionary understanding of what makes us sexually choose, crave, and cherish.

## Search strategies

We prioritized peer-reviewed articles, recent publications (last 10 years), and key older studies to ensure a comprehensive review of both foundational and cutting-edge research. To identify relevant literature, we conducted systematic searches across multiple databases, including PubMed, Google Scholar, Web of Science, and Semantic Scholar. Our search strategy combined key terms such as “socio-sexual”, “social behavior network “, “prenatal sex steroids”, “mammalian sexual behavior”, “sensory pathways”, “pheromones and social behavior”, “mammalian sexual brain”, “neuroendocrine regulation of social behaviors”, “hormonal control of mate choice”, “pair bonding”, “sex differences in the neuroendocrine brain”, and “mate preference test”.

## Data Availability

No datasets were generated or analyzed during the current study; therefore, data sharing is not applicable.
